# Patient Engagement in Research Design and Implementation: Moving From Infancy to Adolescence

**DOI:** 10.1093/geroni/igaa057.2855

**Published:** 2020-12-16

**Authors:** Carol Geary, Martina Roes, Katherine Abbott

## Abstract

Patient (also referred to as User or Older Adult) engaged research is a unique approach to research design, implementation, and dissemination. The practice of engaged research, in its broader sense, involves service recipients, caregivers, clinicians, and other stakeholders in prioritization of research questions, approach, and practice translation to ensure strong stewardship of funds, valid and reliable methods, and practical application. Patient engaged research also aligns with tenets of person-centered care in the inclusive nature and the expectation that the practice will improve the processes and the outcomes of research. Likewise, GSA’s focus on diversity of thought through interdisciplinary work strongly aligns with inclusion of patient and stakeholder voices in the performance of interdisciplinary research. Although patient and stakeholder engagement in research is a new approach for many, globally, funders increasingly require evidence of “meaningful” engagement in project proposals. In the United States, the Patient-Centered Outcomes Research Institute (PCORI) is well-known for funding engaged research and the National Institute on Aging (NIA) has demonstrated use of “collaborative” methods central to patient engagement within the IMPACT Collaboratory. Patient engagement literature, while growing, does not yet provide adequate guidance for replication of current or development of new approaches to patient engagement in research. Therefore, the purpose of this symposium is to frame patient engagement through a historic lens (Roes) and discuss the ethics of engagement (O’Sullivan). In addition, we will share program outcomes from three patient engagement programs: the Leading Age LTSS Center, Rural Patient & Stakeholder Engagement, and Sages in Every Setting. Patient/Person Engagement in Research Interest Group Sponsored Symposium.

